# Optical and Structural Properties of Anisotropic ZnS Nanoparticle Suspensions

**DOI:** 10.1021/acs.langmuir.4c03164

**Published:** 2024-10-16

**Authors:** Naama Gatenio, Sofiya Kolusheva, William Chèvremont, Shachar Moskovich, Dhanush Patil, Kenan Song, Yuval Golan

**Affiliations:** 1 Department of Materials Engineering, Ben-Gurion University of the Negev, Beer-Sheva 8410501, Israel; 2 Ilse Katz Institute for Nanoscale Science and Technology, Ben-Gurion University of the Negev, Beer-Sheva 8410501, Israel; 3 ESRF − The European Synchrotron, 71 avenue des Martyrs, CS40220, 38043 Grenoble Cedex 9,France; 4 School of Environmental, Civil, Agricultural, and Mechanical Engineering (ECAM), College of Engineering, University of Georgia, Athens, Georgia 30602, United States

## Abstract

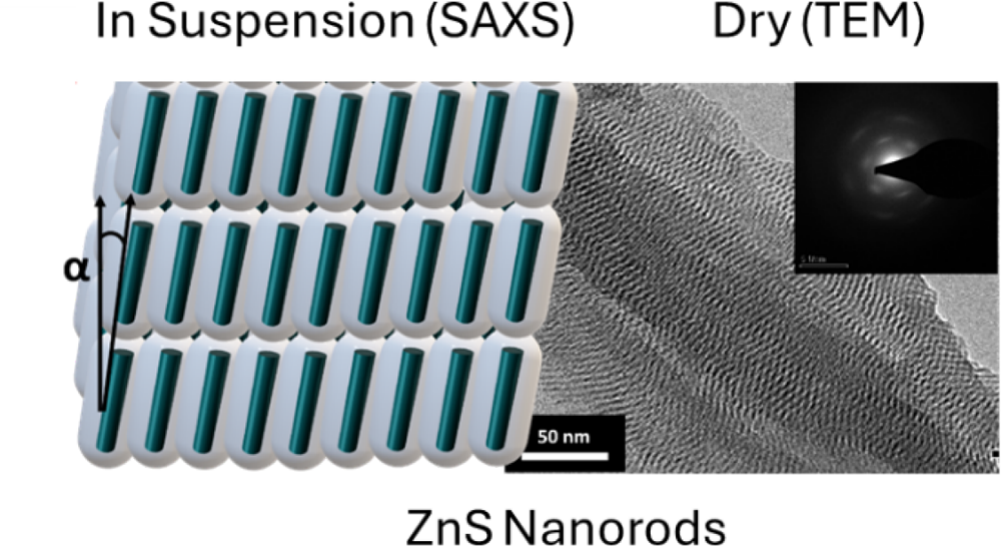

We studied the optical and structural properties of highly ordered arrays of surfactant-capped ZnS nanowires (NWs) and nanorods (NRs) in organic suspensions. The photoluminescence (PL) emission measured under different concentrations and postsynthesis washing cycles interestingly showed increasing emission upon decreasing nanoparticle (NP) concentration. Synchrotron small angle X-ray scattering measurements elucidated the liquid-crystal-like structure of the NPs in suspension under different concentrations and temperatures. The NWs are stacked in a simple structure with a hexagonal cross-section, whereas the structure of the NRs is more complex, resembling a smectic-c liquid crystal, and shows unusual thermal expansion versus temperature. The results point out that a certain amount of bound surfactant must be present on the NP surface to maximize the PL intensity.

## Introduction

There has been increasing interest in nanoparticle (NP) suspensions in recent years due to their unique properties and a broad range of applications. Dispersing NPs in various liquids often results in enhancement of the thermal, electrical, optical, chemical, biochemical, and mechanical properties of the NPs or the suspending liquid, rendering the suspensions scientifically interesting and technologically useful. NP suspensions have been finding applications in important areas of science and technology such as diagnostics and biosensing,^1−3^ energy conversion,^1,4,5^ environmental studies,^6,7^ and catalysis.^2,8^ Of particular interest are ordered suspensions of anisotropic NP,^9^ where the inherent anisotropy of the elements comprising the superstructured arrays introduces an array of physical and optical effects that cannot be attained from isotropic NPs. Commonly referred to as nanofluids in the respective scientific communities, NP suspensions are also increasingly used in heat management^1,10^ and tribological applications.^11,12^

Surfactants play an important role in the colloidal synthesis of the nanocrystals. There are several roles of the surfactant; the most important is as a capping agent that adsorbs onto the surface of the developing seed, thus restricting its size. Additionally, surfactants can serve as dispersing agents that inhibit agglomeration.^13−16^

The use of xanthate salt as a single precursor for both the anion and cation source in synthesis of metal sulfide NP, coupled with alkylamine surfactants used as both capping agents and solvents, has been shown to be useful for obtaining shape and size controlled nanomaterials with high uniformity.^17−20^ A study by Belman et al. on alkylamine surfactants showed that exposure to CO_2_ results in a transformation to alkylammonium alkylcarbamate (AAAC) molecular pairs.^21^ Addition of small concentrations of AAAC to the colloidal synthesis of ZnS nanowires (NWs) coated with octadecylamine (ODA) reduces the aspect ratio, resulting in powder samples of highly uniform surfactant-coated ZnS nanorods (NRs), which were assembled into 2D and 3D ordered arrays.^22−24^

While there has been great interest in applications of NP suspensions,^1−12^ much less has been reported on the ordering of NPs in suspension and their assembly into ordered arrays within the suspending liquid. While small angle X-ray scattering (SAXS), specifically when carried out using ultrahigh brilliance synchrotron X-ray beams, is the most useful technique for studying positional order in NP suspensions,^25^ it has been mainly used for determination of particle size, shape, and concentration.^26^ An interesting and potentially useful application of ordered NPs suspended in polymers and polymer blends is the preparation of advanced “inks” for 3D printing. This emerging technology holds potential for additive manufacturing of advanced materials with tailor-designed optical, mechanical, and magnetic properties.^27^

Belman and co-workers studied the structural properties of ZnS NPs in powder form,^23,24^ as well as at the air–water interface in the form of Langmuir films.^22^ However, the structural properties of these highly ordered anisotropic ZnS NPs in suspension have not been studied to date. In this work, we present a structural and optical study of highly ordered ODA-coated ZnS NRs and NWs suspended in chloroform, elucidating the liquid-crystal-like superstructures these NPs form in the suspension state.

## Experimental details

### Materials

ODA (Aldrich, ≥99.0% (GC)), potassium ethyl xanthogenate (Aldrich, 96%), and zinc perchlorate hexahydrate (Aldrich, 96%), were used as received. ODA was stored under a vacuum in a desiccator. Methanol (Gadot, absolute) and chloroform (Frutarom, AR) were used for all of the experiments. Deionized water (DIW) (resistivity of 18.2 MΩ cm) was obtained from a Millipore filter system.

### Nanoparticle Synthesis

The NPs were prepared by using a modified synthesis based on the method of Pradhan et al.,^19,28^ i.e., heating a preprepared xanthate salt powder in a molten surfactant that is functioning as both the surfactant and solvent of the synthesis. Zinc-ethylxanthate (Zn(SSCOC_2_H_5_)_2_) powder was prepared by dissolving 3 g of potassium ethyl-xanthogenate in DIW and separately dissolving 3.48 g of zinc perchlorate hexahydrate in DIW. The two solutions were mixed for 5 min to form the zinc xanthate salt, washed with DIW, centrifuged three times, and dried in a vacuum. The reaction was carried out in a silicone oil bath on a hot plate inside a simple glass test tube with a magnetic stirrer under nitrogen flow. For surfactant-coated ZnS NW synthesis, 0.08 g of Zn-ethylxanthate was added and dissolved into 1.53 g of molten clean ODA. The nucleation stage was carried out at 110 °C for 5 min, resulting in a turbid, yellowish solution, followed by reaction at 130 °C for 60 min. For surfactant-coated ZnS NR synthesis, 1.53 g of pure ODA was exposed to air in the lab for 4 days, allowing it to react with ambient carbon dioxide gas to partially form octadecylammonium octadecylcarbamate (OAOC) molecular pairs.^21^ The nucleation stage was carried out at 105 °C for 5 min followed by reaction at 130 °C for 8 min. For both NWs and NRs, the reaction was stopped by adding methanol into the test tube that was drawn out of the silicone oil bath. Then the solution was poured into a 50 mL test tube filled with methanol and centrifuged at 2500 rpm for 5 min. Washing was carried out by adding 5 mL of chloroform to the test tube followed by 40 mL of methanol. The mixture was centrifuged three times and finally dried in a vacuum. The final product was obtained as a white powder.

### Characterization methods

Optical transmission measurements of NP suspensions were performed by using a double-beam Jasco V-530 UV–vis spectrophotometer. Clean solvent was used for the background spectra. Photoluminescence (PL) measurements were performed using a Horiba Fluorolog 3. The suspension samples were held in a quartz cuvette equipped with a PTFE screw cap. Excitation was achieved by using a xenon lamp at 265 nm. Lifetime measurements were performed by using a 280 nm LED for excitation.

Thermogravimetric analysis (TGA) measurements were performed using a Thermo Scientific TGA Q500 instrument in a N_2_ atmosphere. The NP powders were held in an alumina crucible and heated to 1000 °C with heating rate 10 °C/min.

Transmission electron microscopy (TEM) samples were prepared by placing three drops of NP suspension in chloroform on a lacey carbon coated copper grid. The grids were laid on filter paper in a plastic sample box to dry. TEM imaging was carried out using an FEI Tecnai G^2^ T-12 TEM operating at 120 kV.

SAXS measurements were performed at the European Synchrotron Radiation Facility (ESRF), Grenoble, France. Measurements were performed at the BioSAXS instrument (BM29 beamline) using a 12.5 keV beam energy and at the TRUSAXS instrument (ID02 beamline) using a 17 keV beam energy. The crystal coherence length was calculated using the Scherrer equation with a geometric factor of *K* = 0.9.

## Results and Discussion

**Figure 1:**
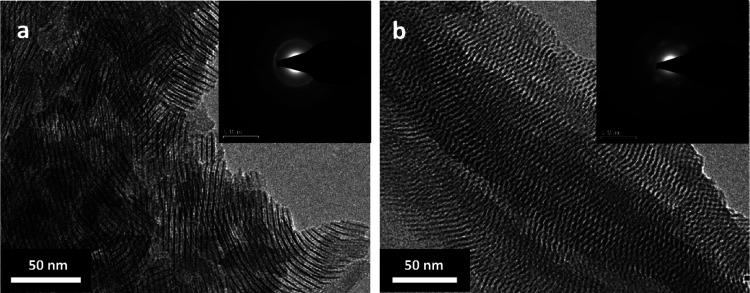
TEM images of dried chloroform suspensions of surfactant-coated ZnS (a) NWs and (b) NRs. Insets show the corresponding electron diffraction patterns.

TEM indicated that the surfactant-coated ZnS NPs obtained are highly ordered and assembled into ordered arrays, as shown in Figure 1. The NPs are ultranarrow, ca. 1 nm in width; the NRs are 5 nm long, while the length of the NWs varies from tens of nanometers to 100 nm. It should be noted that, unlike bulk NP suspensions, samples for TEM represent *dried NP suspensions*; thus, the images do not necessarily represent the structure present in suspensions. However, the TEM images provide a good indication of the general shapes and dimensions of the NPs and can be used for comparing the NW and NR arrays in dry form. The NRs are highly ordered within the array, whereas the NWs are longer than the NRs but are still stacked with typical spacing between them.

TGA was employed to determine the surfactant/particle weight ratio in the NP powders. Both as-synthesized particles and particles after washing cycles were measured. The washing was carried out to remove any excess surfactant. The number of washing cycles examined was one and three times. The analysis is shown in Tables S1 and S2 in the Supporting Information (SI).

**Figure 2:**
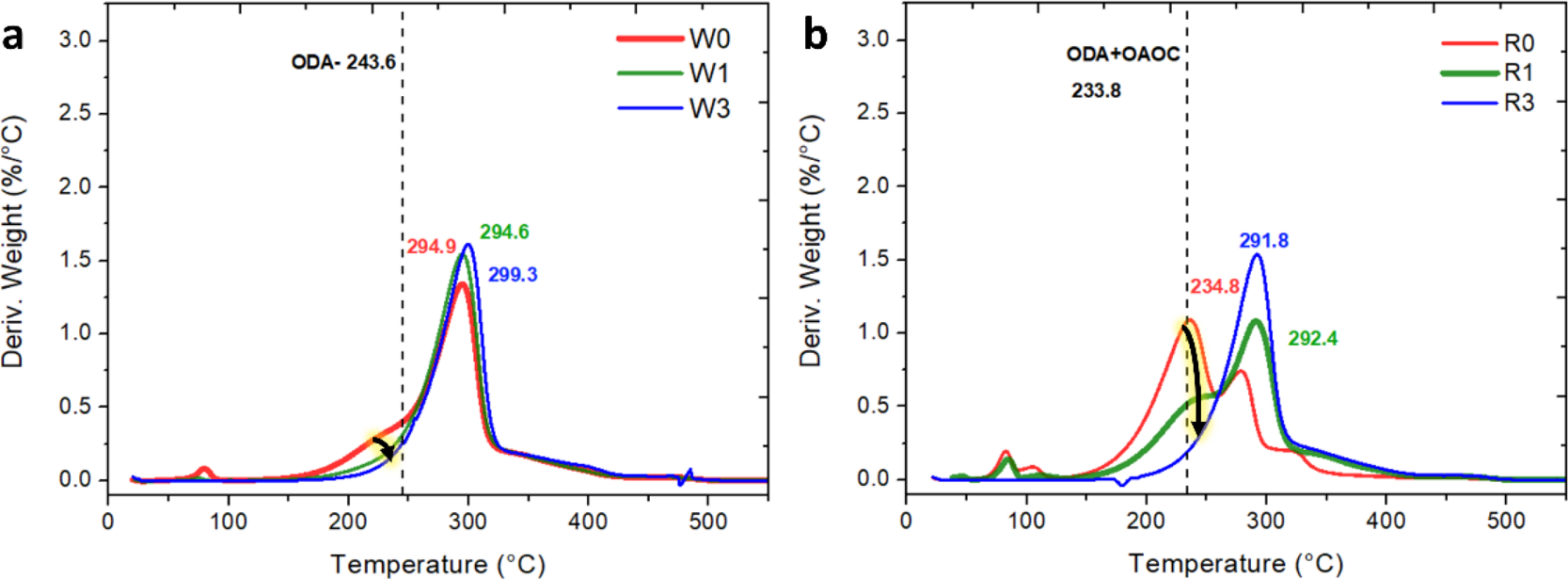
TGA results of ZnS NPs without washing (W0) and after one (W1) and three (W3) washing cycles. (a) NWs and (b) NRs. The dashed vertical lines denote the peak positions of the surfactants in absence of ZnS NPs.

The results in Figure 2 show the mass loss of unwashed and single-cycle-washed NP powders. At 80 °C, the mass loss is related to residual solvent molecules (chloroform and methanol), and the next step of mass loss (up to 250 °C) decreases with the number of washing cycles until it is gone completely after three washing cycles and therefore can be associated with unbound surfactant in the system. The surfactant/ZnS ratio and the residue percentage, which is the remaining inorganic phase (the ZnS cores), decrease with washing cycle, which mean that the washing process results in the loss of ODA. The main peak around 300 °C is related to the bound surfactant. The mass loss percentages around these temperatures increase with the number of cleaning cycles, which mean that the washing process results in the loss of light-bound ODA.

These results are relevant for all types of NPs, NWs, and NRs. However, the latter is characterized by some other features. The presence of OAOC in the surfactant in the NR samples causes a small hydroscopic effect. Water evaporation can be associated with the peak at 110 °C, and the quantity of unbound surfactant in the unwashed and one-time washed NRs is much higher compared to NWs (Figure 2b). The same trend of the peaks around 80 and 110 °C is observed in Figure 2b. The peak at 80 °C is related to solvent molecules, and the peak at 110 °C is assigned to H_2_O molecules adsorbed on the surface of the powder due to the exposure of the surfactant to ambient air. On the other hand, part of OAOC removed during the washing process is free, as indicated by the main peak at 233 °C of the pure OAOC. There are two other peaks that split from the main peak of unwashed NRs powder, and they are related to two different types of OAOC-ZnS bonds associated with the two directions in which an OAOC molecule can bond to the surface of a ZnS NR: on the perimeter and the top bases. After one washing cycle, a certain amount of surfactant is removed, resulting in a tighter structure with stronger bonds.

### Optical Properties

**Figure 3:**
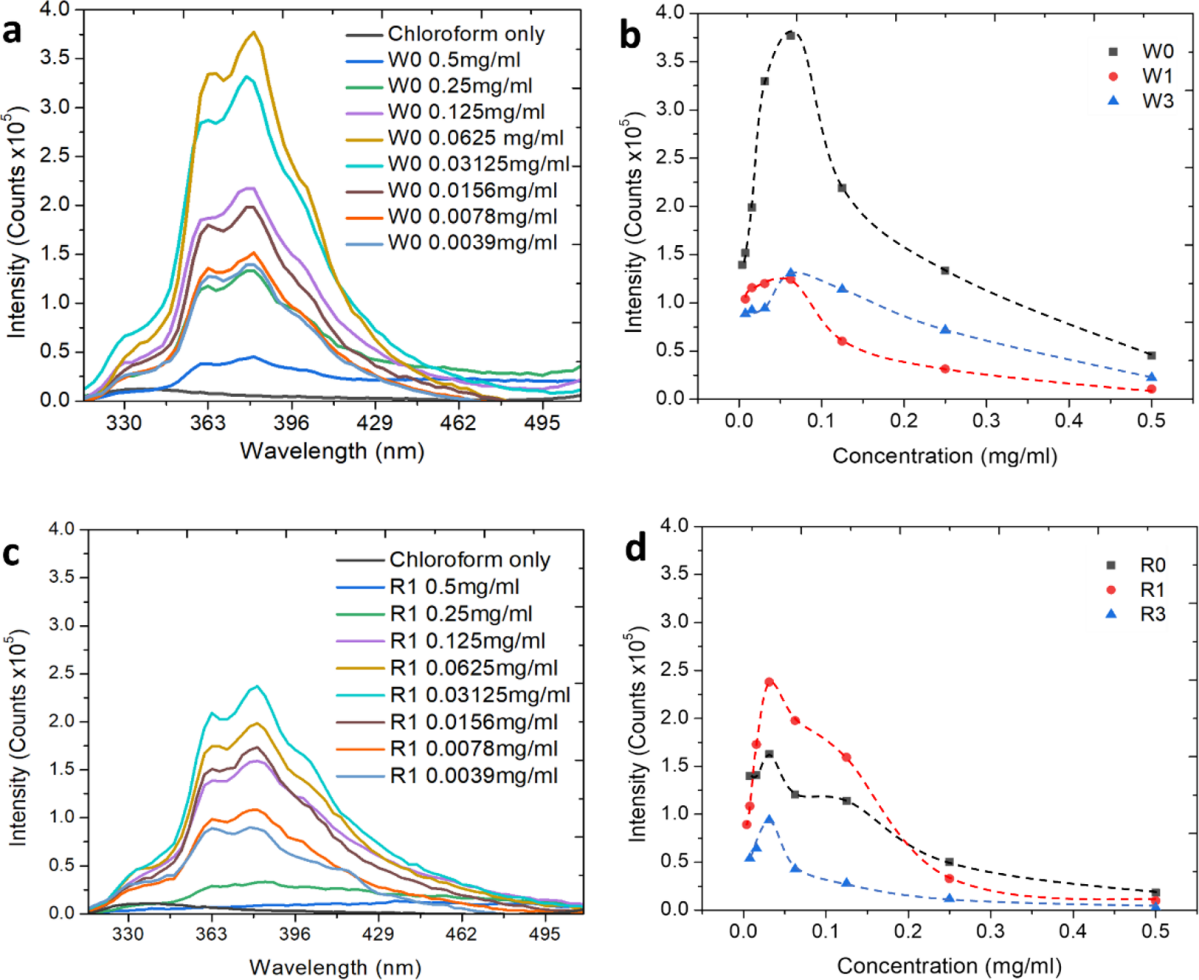
PL measurements of ZnS NPs in chloroform suspension, in different concentrations and number of cleaning cycles. Excitation-265 nm. (a) NWs without washing and (b) PL peak intensity as a function of concentration, of wires without washing, and after one and three cleaning cycles. (c) NRs after one cleaning cycle and (d) PL peak intensity as a function of concentration for rods without washing and after one and three cleaning cycles.

For determination of the excitation wavelength, spectroscopy analysis of nanoparticles in the UV–vis range was performed. The transmission spectra obtained for both NWs and NRs in chloroform suspensions at different concentrations are shown in Figure S1. The results show that the NPs strongly absorb light in the UV region, with a maximum at 280 nm. The position of the maximum absorption wavelength is independent of the concentration of the sample. The PL experiment shows that the emission in the 300–400 nm region was demonstrated by irradiating samples with various wavelengths in the 250–300 nm range, where the maximal emission was observed upon irradiation with 265 nm. Both types of particles showed similar, but not identical, emission spectra, as can be seen in Figure S2. This fact, as well as the presence of several peaks in the overall spectrum, indicates the presence of a few types of electron transitions that give rise to PL properties. These can be explained by the presence of various defects, in particular, sulfur vacancies.^29,30^ There is also a general decrease in the PL properties in the NR samples, which may point to the process of self-quenching or the absence of the required length of the conjugated cascade, as in the case of the NWs. The PL of these particles was also investigated depending on the concentration of particles in the suspension and the number of cleaning cycles after synthesis to remove unbound surfactant, which showed interesting PL emission behavior. The examined cleaning cycles were zero cleaning cycle (unwashed) and one and three cleaning cycles, and the results are shown in Figure 3. The maximal PL emission was obtained for the unwashed NWs and one-cycle washed NRs, which can be seen from Figure 3b,d. The influence of the number of washes and the concentration of particles in suspension provides new and interesting results. As can be seen from Figure 3a,c, both types show identical PL spectra at different concentrations and different numbers of wash cycles. This indicates that the type of electronic excited state does not change depending on the given parameters. But at the same time, the PL intensity undergoes a strong change. Thus, the most active samples are NWs that have not been washed after synthesis, while the most active NR samples are those that have undergone a single wash. Referring to the TGA results in Figure 2, the unwashed NR powder contains a large amount of free surfactant that causes unwanted separation of the NPs, so the PL emission is lower than that of rods after one wash. In addition, the NRs after three washing cycles do not contain free surfactant at all, pointing out that there is not enough separation between the particles and the PL emission is again lower than that of NRs after one washing cycle. We conclude that the NR powder must contain a certain amount of free surfactant to give the maximal PL emission. The influence of NP concentrations in solution is the most interesting phenomenon in these PL investigations. As can be seen from the presented spectra summarized in Figure 3b,d with increasing concentration, an increase in PL is initially observed (as intuitively follows from the law of dependence of emission intensity on concentration) up to a peak value at a concentration of 0.0625 mg/mL for NWs and 0.0313 mg/mL for NRs. At concentrations above these values, a decrease in the intensity begins. This phenomenon may result from the interparticle interaction and energy transfer between individual particles in the concentrated solutions. This effect is observed in both the direct preparation of suspensions of various concentrations and suspensions prepared by the dilution of a concentrated stock solution from high to low concentrations. The phenomenon of increasing PL intensity upon dilution of the solution is likely to be related to the structural features of the NPs and will be further discussed by considering the structural data.

The changes in PL lifetime with concentration were analyzed, and three lifetimes were observed ranging from picoseconds to tens of nanoseconds (shown in Figure S3). This is associated with several types of electron jumps and several types of vacant orbitals. When studying the lifetime, with dilution to the peak concentration, the proportion of long-lived transitions increases in contrast to short ones. These changes also indicate energy quenching transitions between particles in highly concentrated solutions.

### Structural properties

**Figure 4:**
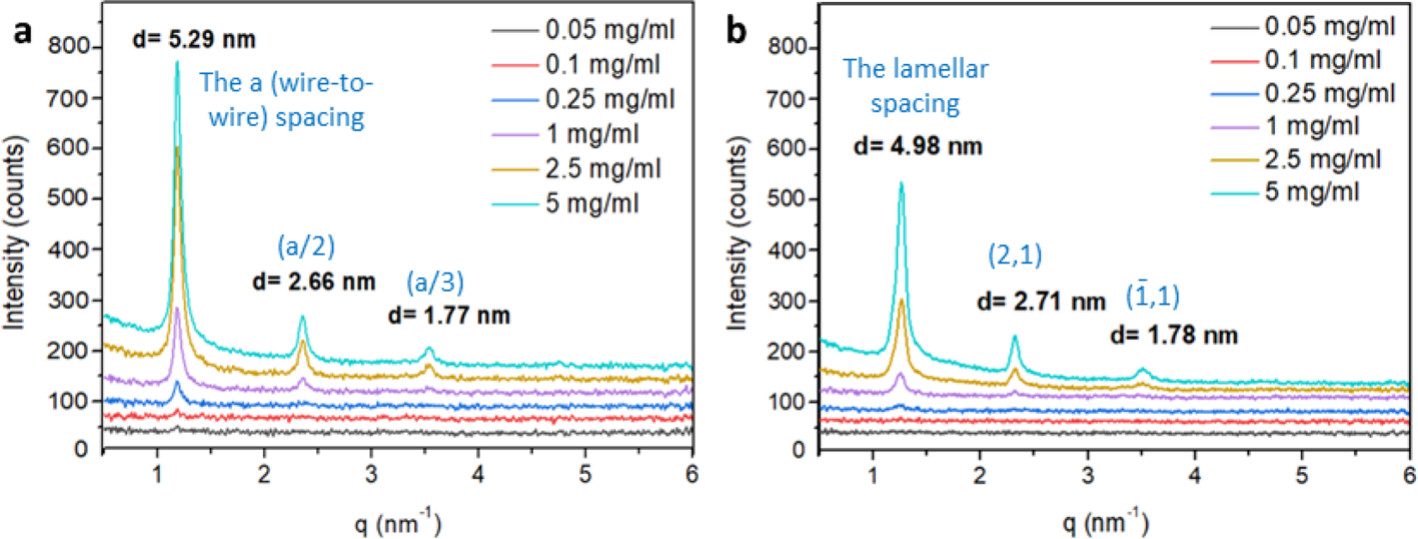
SAXS curves of chloroform suspensions at different concentrations of (a) unwashed NWs. The NW suspension exhibits a fundamental (wire-to-wire, *a-*) spacing, with two subsequent peaks corresponding to the second and third order peaks of the fundamental spacing. (b) Washed one cycle NRs. The first peak obtained from the NRs corresponds to the lamellar spacing, while the second and third peaks correspond to the in-plane structure of the tilted NRs within the lamellae (Figure 5).

SAXS measurements were employed in an attempt to explain the unusual optical properties of the NP suspensions. Synchrotron radiation was employed to allow for sufficient signal-to-noise ratio despite the ultranarrow dimensions of the NPs and the strong scattering from the suspending organic liquid. Figure 4a presents the SAXS results for different concentrations of unwashed NWs in chloroform suspensions. SAXS was also performed for suspensions washed for one and three cycles for NW suspensions at the same concentrations, and the results showed the same trend (not shown).

The first finding is that the peak position does not change with the concentration. However, the scattering intensity increases with concentration, indicating that the dilution of the chloroform suspension, which strongly affected the optical properties, has no impact on the structure of the NPs for the concentrations studied. The highest intensity is obtained at the highest concentration, 5 mg/mL, and the intensity vanishes to zero upon dilution to the lowest concentration studied, 0.05 mg/mL, due to insufficient signal-to-noise from highly diluted samples.

**Figure 5:**
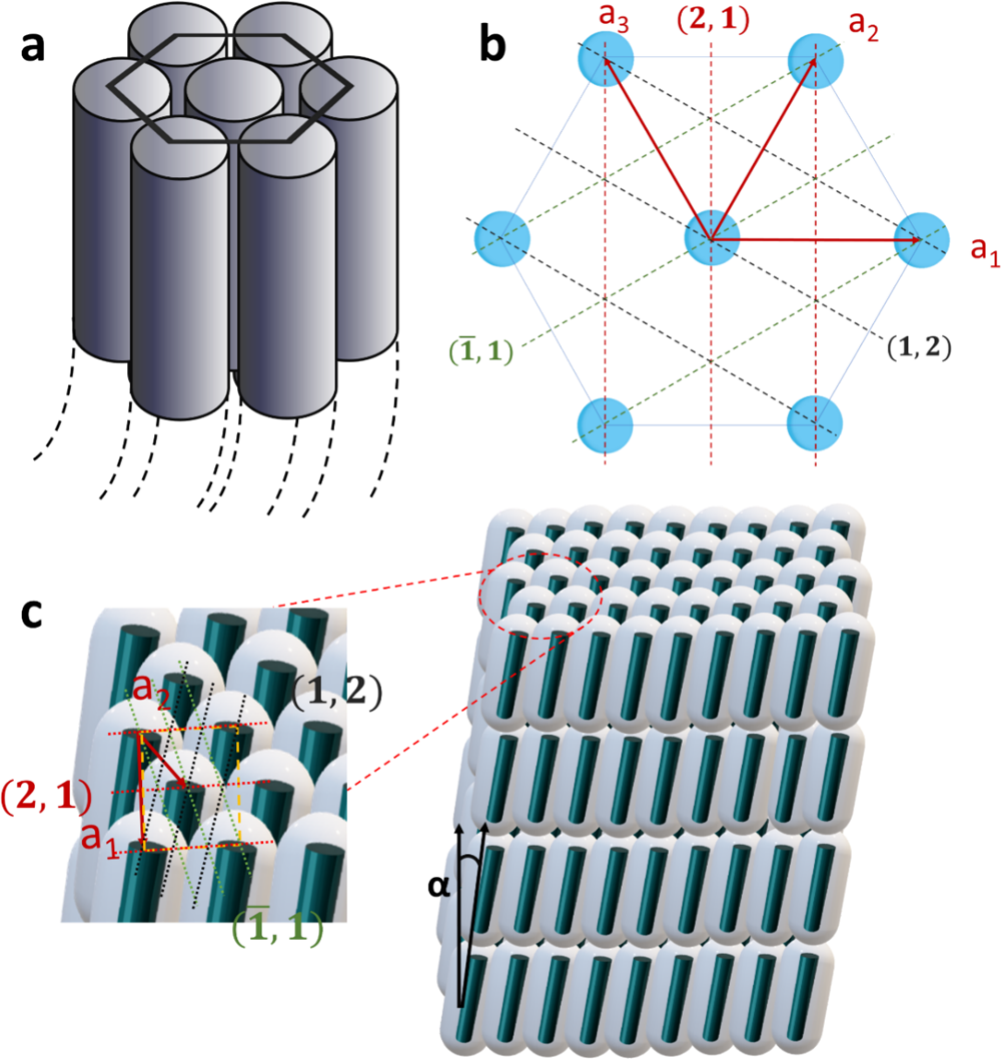
Structural model of NPs in chloroform suspension. (a) Side view of NWs; a simple hexagonal structure. (b) Top view. (c) Structural model of NRs.

The NPs in the suspension spontaneously form an ordered structure that is similar in nature to a liquid crystal. The simplest structure is formed by the NWs, which show a fundamental distance of 5.29 nm (the “*a*” distance) representing the distance between two adjacent surfactant-coated NWs, in agreement with Maiti et al.^31^ The two additional peaks, at *q* = 2.362 and 3.549 nm^–1^, corresponding to *d*-spacings of 2.66 nm (*a*/2) and 1.77 nm (*a*/3), respectively, represent the second and third order peaks of the fundamental distance *a*. This indicates that the NWs are bunched long-sided, as can be seen in the TEM micrograph in Figure 1, to form a hexagonal structure (namely, *a*_1_ = *a*_2_ = *a*_3_), as shown in Figure 5a,b.

It appears that the domains decrease in size as the concentration decreases from 1 mg/mL, which is shown in Figure S3a. In the W0 system, the coherence length is always larger than in the other systems, indicating that washing splits large domains into smaller ones. Coherence length greatly changes with concentration in the W1 and W3 systems, confirming that these systems show a smaller degree of uniformity.

Figure 4b presents the SAXS results for different concentrations of NRs in chloroform suspensions. The SAXS results of the one-cycle-washed NRs shows a first peak at *q* = 1.261 nm^–1^ corresponding to *d*_1_= 4.98 nm, a second peak with weaker intensity at *q* = 2.318 nm^–1^ corresponding to *d*_2_= 2.71 nm, and an even weaker peak at *q*= 3.529 nm^–1^ corresponding to *d*_3_= 1.78 nm. The second and third peaks are clearly not high-order peaks of a fundamental distance, as was observed for the ZnS NWs. Given the “ribbon-like” packing observed in TEM for dried samples, it is reasonable to assume that, unlike the NWs, the NRs form a lamellar structure in which the rods are packed perpendicular to the lamellar thickness, as shown in Figure 1. The distance of *d*_1_ = 4.98 nm corresponds well to the length of the NRs, namely, the thickness of the lamellae. However, because at least one terminal of the rods should be covered with surfactant molecules, it is reasonable to assume that the *d*_1_ spacing obtained is due to the tilt of the rods within the lamellae, resembling a smectic-c liquid crystal, as depicted in Figure 5c. An estimation for the tilt angle can be obtained if we compare the ribbon-to-ribbon distance observed in the TEM, 5.75 nm,^23,24^ to the spacing *d*_1_ = 4.98 nm obtained for the NR suspensions from SAXS, which gives a tilt angle of at least α = 29.6° from the vector normal to the lamellae. This angle may increase depending on the amount of surfactant adsorbed onto the two c-termini of the wurtzite NRs.

Reinforcement for the tilted nature of the NRs within the lamellae is obtained from the positions of the *d*_2_ and *d*_3_ peaks, which arise from the in-plane structure of the NRs. The spacing of *d*_2_ at 2.71 nm matches reasonably well with the second order of the fundamental spacing obtained for the NWs. This implies that the NRs within each lamella are tilted within the (2,1) planes, and therefore, tilt does not affect the spacing of these *d*_2_ planes. However, this tilt is affecting the spacing of the (1,2) and (−1,1) planes, which are symmetrical on both sides of the (2,1) planes, giving rise to a decrease in their spacing to *d*_3_ = 1.78 nm.

In conclusion, the NRs within each lamella are arranged in an in-plane structure corresponding to a distorted or “compressed” hexagonal (centered rectangular) structure with in-plane vectors *a*_1_ = 5.40 nm and *a*_2_ = 3.56 nm, and the angle between them is φ = 40.7°. Interestingly, the value of *a*_2_ = 3.56 nm is in good agreement with the 3.59 nm spacing obtained from SAXS for the same ZnS NRs, yet in powder form [see ref (24), Figure 3b].

For the R1 system, the coherence length increases with the dilution, which implies that the system is less dense, and for the R3, the opposite trend is observed (Figure S4). The R0 system does not show a clear trend of the coherence length, but the values are always higher than for the other systems, like in the wire case. This also implies a trend of smaller domains as a function of the number of wash cycles.

**Figure 6:**
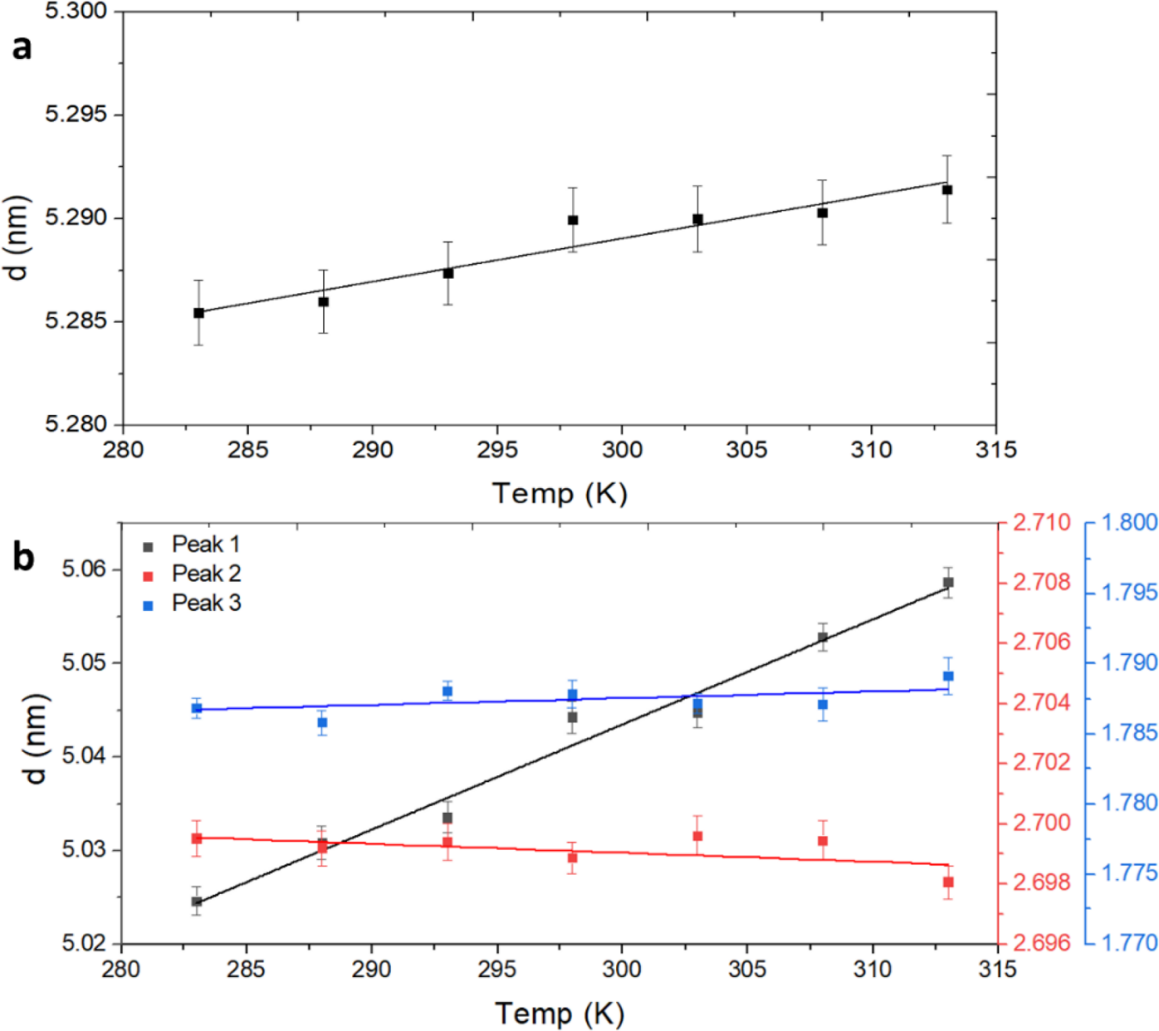
ZnS NPs in a chloroform suspension. The *d*-spacing as a function of temperature of 5 mg/mL chloroform suspension of (a) the first order peak in SAXS of ZnS NWs and (b) the first, second, and third peaks in SAXS of ZnS NRs.

As expected, the temperature has a pronounced effect on the *d*-spacing obtained for the NP arrays. Figure 6a,b shows the position of the first peak (*d*_1_) as a function of temperature for a 5 mg/mL chloroform suspension of ZnS NWs and NRs, respectively.

The subtle shift in peak position for the NW suspension, as seen in Figure 6a, behaves linearly with temperature, which allowed us to extract a linear thermal expansion coefficient of α_1, w_ = 3.95 × 10^–5^ K^–1^ for the wires in the radial direction, namely, perpendicular to the long axis of the wires. As expected, the linear thermal expansion coefficients for the two high-order peaks are very similar. The thermal expansion coefficients were calculated for each *d*-spacing using linear fitting (the calculation is provided in the SI). In the case of NW suspensions, the thermal expansion coefficients obtained were as follows: α_1, *w*_ = 3.95 × 10^–5^ K^–1^, α_2, *w*_ = 5.3 × 10^–5^ K^–1^, and α_3, *w*_ = 4.7 × 10^–5^ K^–1^. These coefficients are quite similar, which confirm that the second and third peaks are high-order peaks of the first and the NW system is based on a single distance.

Unlike the NWs, which exhibited a single fundamental characteristic distance, the structure of the NRs is more complex, with one peak, *d*_1_, corresponding to the lamellar stacking of the NRs and two peaks, *d*_2_ and *d*_3_, arising from the in-plane structure of the NRs within the lamellae, which is shown in Figure 6b. In the case of NR suspensions, the thermal expansion coefficients obtained were as follows: α_1, *r*_ = 2.23 × 10^–4^ K^–1^, α_2, *r*_ = −1.12 × 10^–5^ K^–1^, and α_3, *r*_ = 2.67 × 10^–5^ K^–1^. The thermal expansion coefficients α_2, *r*_ and α_3, *r*_ are of the same order as the thermal expansion coefficient obtained for the NWs, supporting the assignment of these directions perpendicular to the long axis of the NWs/NRs. However, the α_1, *r*_ thermal expansion coefficient of the rods in the axial direction is an order of magnitude larger, supporting its assignment as corresponding to the lamellar spacing *d*_1_ of the NRs. Interestingly, the negative sign of α_2, *r*_ suggests that the complex response of the NRs to temperature involves straightening of the NRs within the lamellae, namely, decreasing the angle α between the long axis of the NRs and the vector normal to the lamellar planes. This, in turn, relaxes the distortion of the compressed hexagonal structure, resulting in an increase in *d*_3_ while that of *d*_2_ is simultaneously decreasing.

In summary, with increasing temperature, the tilt of the NRs decreases, and the hexagonal structure of the NRs within the lamellae becomes less distorted. These findings are in line with the TGA results, which showed a larger surfactant/ZnS ratio in the NRs compared with the NWs, probably due to a high concentration of surfactant molecules between the lamellae in the case of NRs, giving rise to the significantly higher value of α_1, *r*_.

## Conclusions

The sample preparation conditions of chloroform suspensions of ZnS NRs and NWs have a strong effect on their optical and structural behavior. The optimal excitation wavelength of the NPs in the chloroform suspension was found to be 265 nm. The PL emission is affected by the number of cleaning cycles of the NPs (affecting the surfactant/ZnS ratio) and the concentration of the NPs in the suspension. A certain amount of bound surfactant and free surfactant is necessary for obtaining maximal PL maximal emission. For the NRs, too much surfactant or not enough of it causes aggregation, which in turn leads to decreased PL emission. Dilution of the suspension significantly improves the PL emission due to weaker interparticle interactions and decreased energy transfer between particles compared to more concentrated suspensions up to a weight concentration of about 0.05 mg/mL. From this concentration, the PL emission of more diluted suspensions is decreasing due to the decrease in the amount of the light-emitting substance. The structural properties of the chloroform suspensions were studied using SAXS. Unfortunately, concentrations below 0.1 mg/mL were not measurable using SAXS. Despite the Extremely Brilliant Source (EBS) available at the ESRF since August 2020, the ultrahigh brilliance and fast detection capabilities could not be utilized in full due to radiation damage and strong absorption of the suspending liquid, which required significant attenuation of the beam.

As expected, dilution decreases the SAXS signal (smaller amount of crystalline material), while the peak positions remain unchanged. The NWs assemble into a liquid-crystal-like structure with hexagonal symmetry, while the NRs assemble into smectic-c-like liquid crystals with a centered rectangular in-plane structure within the smectic lamellae. The number of cleaning cycles also affects the distribution of the NP domain size. The more cleaning cycles are carried out, the less uniform is the NP suspension in terms of domain size for both NWs and NRs, as seen from the coherence length results. The thermal expansion of NR assemblies is more affected by the temperature compared to that of NW assemblies. The NWs showed a single fundamental spacing that increases upon heating. Interestingly, in the case of NRs, each of the *d*-spacings obtained showed a different behavior upon heating—in both magnitude and sign of thermal expansion.

## Supporting Information Available

Optical transmission spectra, PL at different excitation energies, TGA analysis for NWs, TGA analysis for NRs, lifetime measurements, coherence lengths obtained from SAXS peak broadening, and thermal expansion calculation (PDF)
