# Supplementary material for: Optical and Structural Properties of Anisotropic ZnS Nanoparticle Suspensions
Source: Langmuir. 2024 Oct 16;40(43):22982–9. doi: 10.1021/acs.langmuir.4c03164 (PMC11526353; doi:10.1021/acs.langmuir.4c03164)
Supplement: Supplementary file 1 — la4c03164_si_001.pdf [file la4c03164_si_001.pdf]

Optical and Structural Properties of Anisotropic ZnS Nanoparticle Suspensions

Supporting Information

Naama Gatenio,[1,2] Sofiya Kolusheva,[2] William Chèvremont,[3] Shachar Moskovich,[1,2] Dhanush Patil,[4] Kenan Song,[4] and Yuval Golan*[1,2]

[1] Department of Materials Engineering, Ben-Gurion University of the Negev, Beer-Sheva 8410501, Israel

[2] Ilse Katz Institute for Nanoscale Science and Technology, Ben-Gurion University of the Negev, Beer-Sheva 8410501, Israel

[3] ESRF – The European Synchrotron, 71 avenue des Martyrs, CS40220, 38043 Grenoble Cedex 9, France

[4] School of Environmental, Civil, Agricultural, and Mechanical Engineering (ECAM), College of Engineering, University of Georgia, Athens, GA, 30602 USA

* Corresponding author. E-mail: ygolan@bgu.ac.il

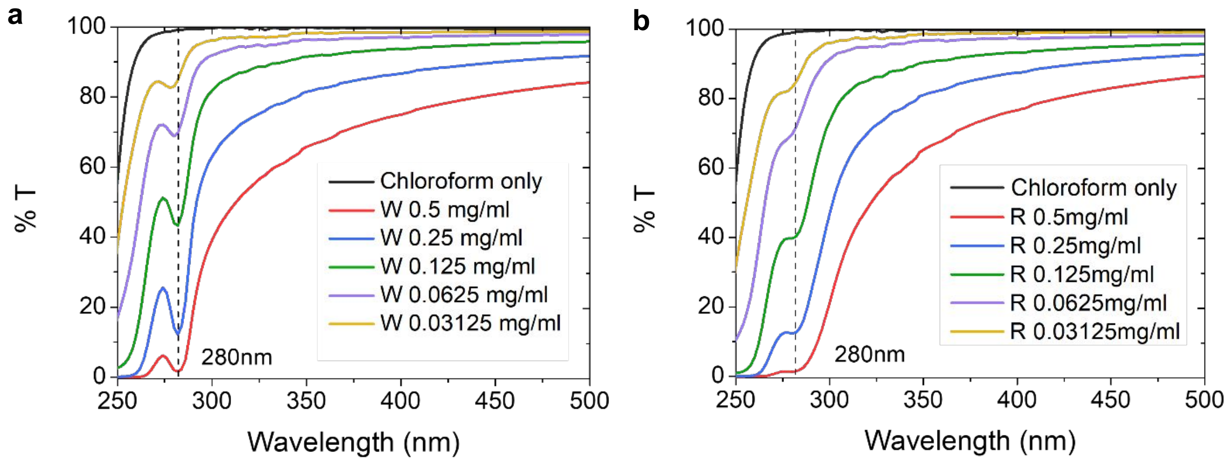

Figure S1. (a,b) Optical transmission of chloroform suspensions of ZnS nanowires (NWs) and nanorods (NRs) at different concentrations.

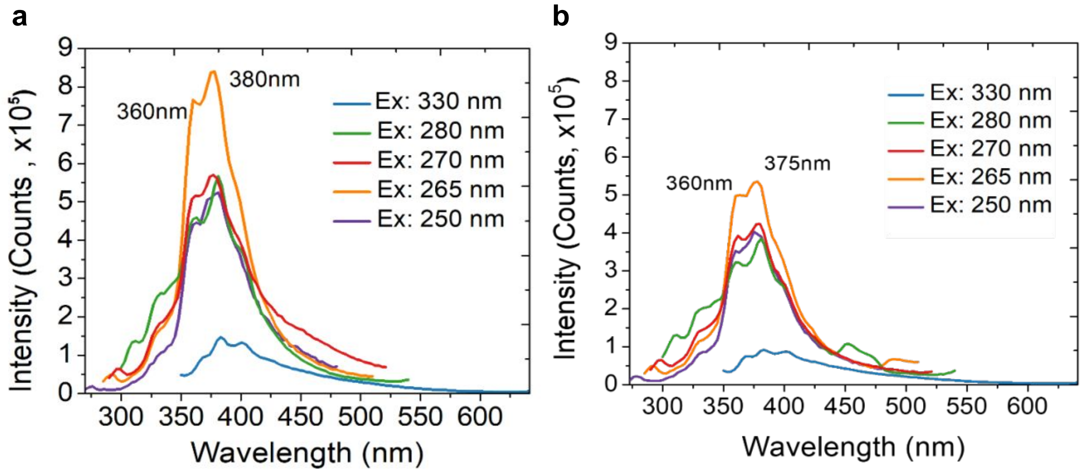

Figure S2. Photoluminescence (PL) emission with different excitation wavelengths for a highly dilute (0.01mg/mL) chloroform suspension of (a) NWs and (b) NRs.

Table S1. TGA analysis for NWs.

	Weight loss, Tm~ 80°C (%)	Weight loss, Tm~ 220°C (%)	Weight loss, Tm~ 245°C (%)	Weight loss, Tm~ 300°C (%)	Weight loss, Tm~ 350°C (%)	Residue (%)	Surfactant / ZnS ratio
**W0**	2.56	14.63	-	61.30	12.14	7.85	11.21
**W1**	0.167	-	-	78.14	11.14	9.91	9.00
**W3**	-	-	-	76.16	12.21	9.65	9.15
**ODA**	-	-	100	-		-	-

Table S2. TGA analysis for NRs.

	Weight loss, Tm~ 80°C (%)	Weight loss, Tm~ 110°C (%)	Weight loss, Tm~ 236°C (%)	Weight loss, Tm~ 290°C (%)	Weight loss, Tm~ 325°C (%)	Residue (%)	Surfactant / ZnS ratio
**R0**	4.60	2.53	57.73	22.64	7.75	4.28	20.6
**R1**	2.66	1.73	30.86	46.2	10.98	7.56	10.4
**R3**	-	-	-	67.44	17.63	13.08	6.5
**OAOC**	2.39	1.28	96.53	-		-	-

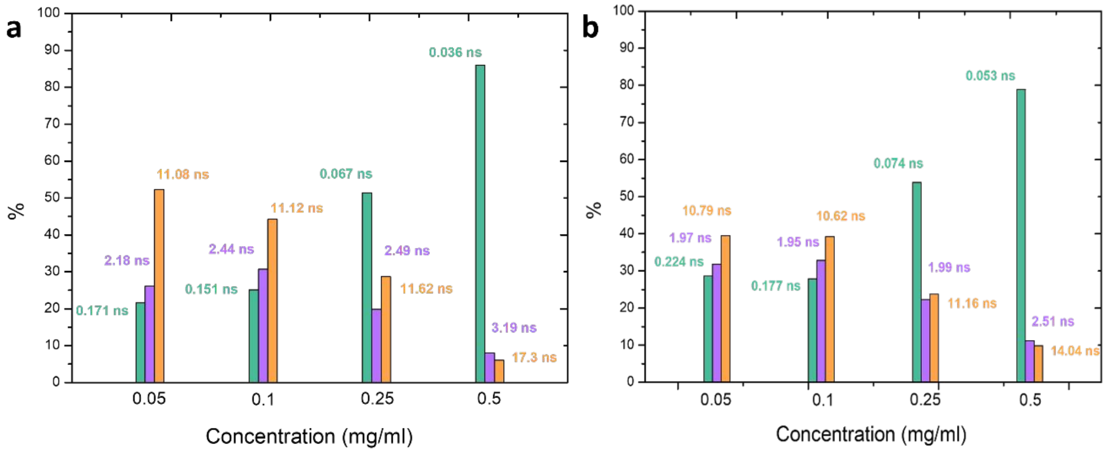

Figure S3. Lifetime distribution of different concentrations in chloroform suspensions of (a) NWs and (b) NRs. Ex-280 nm, Em-380 nm. The Y axis represents the percentage of each lifetime in total value.

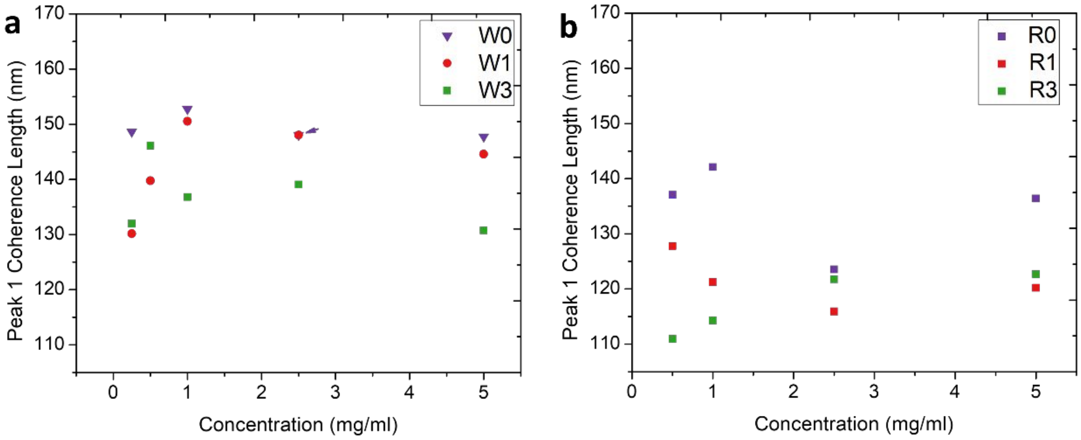

Figure S4. Coherence length obtained from the first peak of the SAXS results, plotted vs. concentration. (a) NWs sample. the arrow is pointing to a W0 point (purple) that is hidden under the W1 point (red) due to same coherence length values for that concentration. (b) Nanorod sample.

Thermal expansion

Thermal expansion was evaluated from temperature resolved SAXS measurements of nanoparticle suspensions in chloroform. The thermal expansion coefficient α was calculated from the slope obtained by linear fitting the plot of d-spacing vs. temperature, as follows:

$$l_{f(T)} = l_0 \cdot \alpha \cdot \Delta T + l_0 \rightarrow \alpha = \frac{Slope}{l_0}$$
